# Ivermectin metabolites reduce *Anopheles* survival

**DOI:** 10.1038/s41598-023-34719-2

**Published:** 2023-05-19

**Authors:** Kevin C. Kobylinski, Phornpimon Tipthara, Narenrit Wamaket, Sittinont Chainarin, Rattawan Kullasakboonsri, Patchara Sriwichai, Siriporn Phasomkusolsil, Borimas Hanboonkunupakarn, Podjanee Jittamala, Renia Gemmell, John Boyle, Stephen Wrigley, Jonathan Steele, Nicholas J. White, Joel Tarning

**Affiliations:** 1 Department of Entomology, Armed Forces Research Institute of Medical Sciences, 315/6 Rajvithi Road, Bangkok, 10400 Thailand; 2 Mahidol Oxford Tropical Medicine Research Unit, Faculty of Tropical Medicine, Mahidol University, 420/6 Rajvithi Road, Ratchathewi, Bangkok, 10400 Thailand; 3 Mahidol Vivax Research Unit, Faculty of Tropical Medicine, Mahidol University, 420/6 Rajvithi Road, Ratchathewi, Bangkok, 10400 Thailand; 4 Department of Medical Entomology, Faculty of Tropical Medicine, Mahidol University, 420/6 Rajvithi Road, Ratchathewi, Bangkok, 10400 Thailand; 5 Department of Clinical Tropical Medicine, Faculty of Tropical Medicine, Mahidol University, 420/6 Rajvithi Road, Ratchathewi, Bangkok, 10400 Thailand; 6 Department of Tropical Hygiene, Faculty of Tropical Medicine, Mahidol University, 420/6 Rajvithi Road, Ratchathewi, Bangkok, 10400 Thailand; 7 Hypha Discovery Limited, 154B Brook Drive, Abingdon, OX14 4SD Oxfordshire UK; 8 Centre for Tropical Medicine and Global Health, Nuffield Department of Clinical Medicine, University of Oxford, Old Road Campus, Oxford, OX3 7BN UK

**Keywords:** Translational research, Malaria

## Abstract

Ivermectin mass drug administration to humans or livestock is a potential vector control tool for malaria elimination. The mosquito-lethal effect of ivermectin in clinical trials exceeds that predicted from in vitro laboratory experiments, suggesting that ivermectin metabolites have mosquito-lethal effect. The three primary ivermectin metabolites in humans (*i.e*., M1 (3″-*O*-demethyl ivermectin), M3 (4-hydroxymethyl ivermectin), and M6 (3″-*O*-demethyl, 4-hydroxymethyl ivermectin) were obtained by chemical synthesis or bacterial modification/metabolism. Ivermectin and its metabolites were mixed in human blood at various concentrations, blood-fed to *Anopheles dirus* and *Anopheles minimus* mosquitoes, and mortality was observed daily for fourteen days. Ivermectin and metabolite concentrations were quantified by liquid chromatography linked with tandem mass spectrometry to confirm the concentrations in the blood matrix. Results revealed that neither the LC_50_ nor LC_90_ values differed between ivermectin and its major metabolites for *An. dirus* or *An. minimus.*, Additionally, there was no substantial differences in the time to median mosquito mortality when comparing ivermectin and its metabolites, demonstrating an equal rate of mosquito killing between the compounds evaluated. These results demonstrate that ivermectin metabolites have a mosquito-lethal effect equal to the parent compound, contributing to *Anopheles* mortality after treatment of humans.

## Introduction

Ivermectin mass drug administration (MDA) is a potential new tool for malaria control and elimination. Blood-feeding on ivermectin-treated humans or livestock is lethal to *Anopheles* mosquitoes found in Africa, the Americas, Asia, Southeast Asia, and the South Pacific^1^. In the Greater Mekong Subregion (GMS) malaria vector control is complicated by the outdoor and early evening^2,3^ or late morning^4^ feeding behaviors of many of the important primary and secondary malaria vectors in the region. Several GMS malaria vectors are susceptible to ivermectin at human- and cattle-relevant concentrations, including *Anopheles dirus* s.s.^5–7^, *Anopheles minimus* s.s.^5,6^, *Anopheles sawadwongporni*, *Anopheles campestris*^5^, and *Anopheles epiroticus*^7^.

The impact of ivermectin on *Anopheles* survival when fed on blood from a treated host in vivo consistently exceeds that of when mosquitoes are fed blood spiked with ivermectin in vitro. This discrepancy has been observed from clinical trials with *Anopheles gambiae* in Kenya^8^, and *Anopheles dirus* and *Anopheles minimus* in Thailand^6^. One explanation for this difference is that there are active metabolites of ivermectin, which possess a mosquito-lethal effect. If slowly eliminated ivermectin metabolites impart a mosquito-lethal effect, then this would result in a longer duration of mosquito mortality than previously predicted^5^.

**Figure 1:**
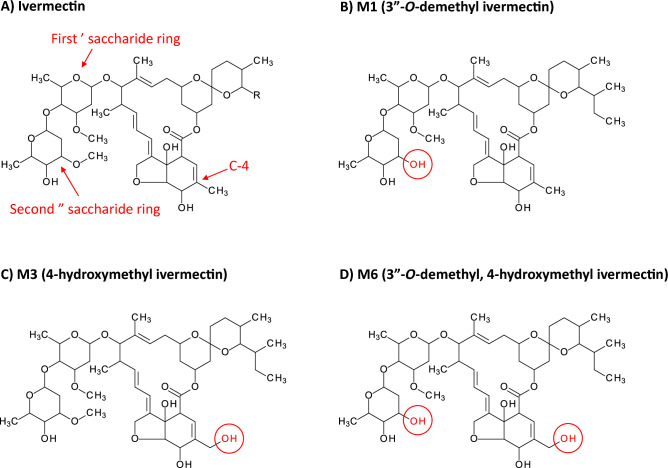
Molecular structure of ivermectin and its primary human metabolites. Molecular structures of (**A**) ivermectin parent compound, (**B**) M1 (3″-*O*-demethyl ivermectin) which has a methyl group removed from the second saccharide ring, (**C**) M3 (4-hydroxymethyl ivermectin) which has an extra hydroxyl group at C-4, and (D) M6 (3″-*O*-demethyl, 4-hydroxymethyl ivermectin) which has both the extra hydroxyl group at C-4 and a methyl group removed from the second saccharide ring. The red circles denote changes in the metabolite structures compared to parent compound (**B**–**D**).

There are three primary metabolites produced in humans at detectable concentrations following ivermectin ingestion; M1 (3″-*O*-demethyl ivermectin), M3 (4-hydroxymethyl ivermectin), and M6 (3″-*O*-demethyl, 4-hydroxymethyl ivermectin) (Fig. 1)^9^. Previous protein crystallography work in a *Caenorhabditis elegans* model with the glutamate-gated chloride (GluCl) ion channel, the invertebrate target of ivermectin, suggested that the first sugar ring is critical to binding to the M2-M3 loop, and consequent activation of the GluCl channel. In addition, the fourth carbon (C-4) of the macrocyclic ring structure was shown to bind to the M2 subunit^10^. Thus, the demethylation of the second sugar ring (*i.e.*, M1 and M6), or hydroxylation of C-4 (*i.e.*, M3 and M6) have the potential to affect mosquito-lethal outcomes.

Here, we assess the mosquito-lethal effect of the three most abundant ivermectin metabolites generated in humans^9^ on *An. dirus*, one of the most ivermectin-tolerant species, and *An. minimus*, one of the most ivermectin-susceptible species^1,5,6^.

## Results

### Mosquito mortality results

Preliminary mosquito feeds produced inconsistent mosquito mortality results (data not shown). To investigate this, aliquots from each mosquito blood meal were analysed by liquid chromatography linked with tandem mass spectrometry (LC–MS/MS) to verify the concentrations of ivermectin and its metabolites. A two to tenfold loss from intended target concentrations was found when analyzing blood meals with LC–MS/MS; ivermectin 53% loss, M1 63% loss, M3 77% loss, M6 88% loss. This random loss is most likely a result from non-specific binding of ivermectin and metabolites to plastic during dilution in phosphate buffered saline (PBS) for bloodmeal preparations. Thus, the concentrations evaluated could not be assessed from the quantities added and only from those measured. Mosquito survival results were therefore linked to LC–MS/MS-quantified ivermectin and metabolite bloodmeal concentrations to derive the LC_50_ and LC_90_ estimates. Mosquito survival results associated with ivermectin or metabolite concentrations below the lower limit of quantification (LLOQ) were excluded from the LC_50_ analyses. Total mosquito mortality was assessed at day 14 post-feeding as the primary pharmacodynamic parameter.

**Figure 2:**
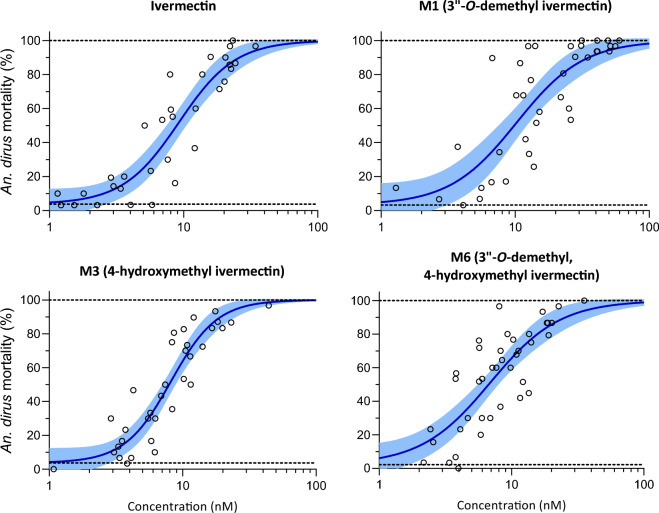
*Anopheles dirus* mortality results when fed ivermectin and metabolites in human blood. Open circles represent cumulative mosquito mortality at 14 days after blood meal ingestion. Solid lines represent the mean concentration–response relationship and the shaded area represents the 95% confidence interval associated with the nonlinear fit. Dashed black lines represent the fixed maximum effect of 100% mortality and the estimated minimum effect associated with baseline mortality observed from control mosquitoes.

**Figure 3:**
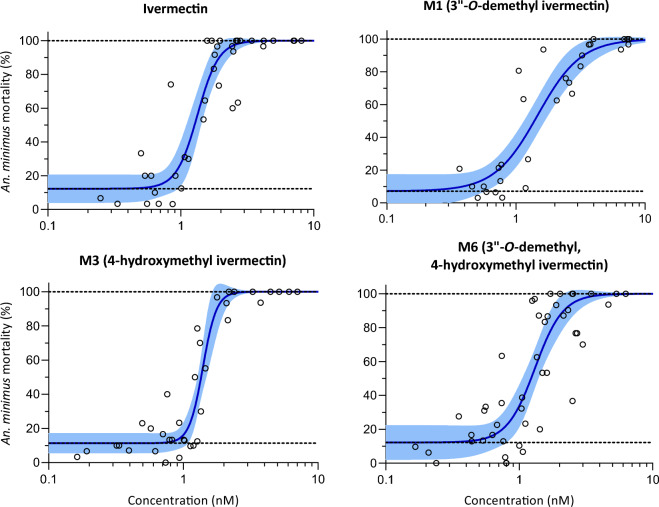
*Anopheles minimus* mortality results when fed ivermectin and metabolites in human blood. Open circles represent cumulative mosquito mortality at 14 days after blood meal ingestion. Solid lines represent the mean concentration–response relationship and the shaded area represents the 95% confidence interval associated with the nonlinear fit. Dashed black lines represent the fixed maximum effect of 100% mortality and the estimated minimum effect associated with baseline mortality observed from control mosquitoes.

Six replicates with a total of 4638 *An. dirus* mosquitoes were used to calculate the LC_50_ and LC_90_ values for ivermectin compounds including: parent compound (n = 1142), M1 (n = 1405), M3 (n = 1203), and M6 (n = 1428). Seven replicates with a total of 4789 *An. minimus* were used to calculate the LC_50_ and LC_90_ values for ivermectin compounds including: parent compound (n = 1284), M1 (n = 1158), M3 (n = 1318), and M6 (n = 1626). All three ivermectin metabolites had mosquito-lethal effects, which were similar to those of the parent compound for both *An. dirus* (Fig. 2, Table 1) and *An. minimus* (Fig. 3, Table 1). The susceptibility of *An. minimus* was substantially lower than *An. dirus* by 5.3- to 6.9-fold depending on the compound (Table 1). Furthermore, ivermectin 10-day-LC_50_ for *An. dirus* was 9.68 [7.97–11.66] ng/mL and 1.42 [1.22–1.59] ng/mL for *An. minimus,* which are 3.6-fold and 3.8-fold higher than that previously reported from clinical trial results (2.66 [2.49–2.83] ng/mL for *An. dirus* and 0.38 [0.37–0.42] ng/mL for *An. minimus*), respectively^6^. This further supports that both ivermectin and its metabolites contribute equally to in vivo mosquito mortality. Similar discrepancies were seen when comparing the time to median mosquito mortality between clinical trial data and in vitro data generated here. The median *An. dirus* survival time was 3 days for clinical data and 10 days for in vitro data at 5 ng/mL of ivermectin. Similarly, the median *An. minimus* survival time was 3 days for clinical data and 10 days for in vitro data at 1 ng/mL of ivermectin (Fig. S16). This was also shown as a right shift of the concentration–response curve when evaluating the median time to death with a dose–response analysis, resulting in an estimated threefold to fourfold higher concentration associated with half of maximum survival time (TC_50_) for in vitro data compared to clinical data (Fig. S16). Additionally, ivermectin and its metabolites showed similar median time to mosquito mortality in the in vitro data generated here (Figs. S17 and S18). This supports further that both ivermectin and its metabolites contribute equally to the overall clinical mosquito mortality associated with ivermectin administration.

**Table 1:** *An. dirus* and *An. minimus* LC_50_ and LC_90_ values for ivermectin and metabolites calculated based on 14 day observation.

Species	Compound	LC_50_ [95% CI] (nM)	LC_90_ [95% CI] (nM)
*An. dirus*	Ivermectin	9.24 [7.54–11.21]	26.70 [19.56–39.12]
	M1	10.24 [7.562–13.43]	36.22 [23.68–62.53]
	M3	8.17 [7.09–9.34]	19.67 [15.35–26.81]
	M6	6.92 [5.45–8.67]	26.07 [17.92–44.19]
*An. minimus*	Ivermectin	1.34 [1.16–1.52]	1.20 [1.64–2.62]
	M1	1.48 [1.21–1.83]	3.46 [2.40–5.07]
	M3	1.40 [1.32–1.52]	1.81 [1.55–2.26]
	M6	1.31 [1.13–1.51]	2.24 [1.61–3.56]

LC_50_ and LC_90_ represent the lethal concentration that kills 50% and 90%, respectively, of either *An. dirus* or *An. minimus* at fourteen days after a blood meal containing ivermectin parent compound, M1 (3″-*O*-demethyl ivermectin), M3 (4-hydroxymethyl ivermectin), or M6 (3″-*O*-demethyl, 4-hydroxymethyl ivermectin).

## Discussion

The mosquito-lethal effects of ivermectin given to human subjects exceed those associated with exposure of *Anopheles* mosquitoes to the same ivermectin concentrations in spiked blood samples. This difference suggests that ivermectin metabolites could contribute substantially to its mosquito-lethal activity. This study confirms this hypothesis. This is the first time that the mosquito-lethal effect of ivermectin metabolites on *Anopheles* survival has been investigated in an in vitro system. The three primary human ivermectin metabolites (M1, M3, and M6) investigated here each have mosquito-lethal effect similar to that of ivermectin parent compound for both *An. dirus* (Fig. 2, Table 1) and *An. minimus* (Fig. 3, Table 1), two primary malaria vectors in the GMS. Furthermore, the ivermectin 10-day-LC_50_s for *An. dirus* and *An. minimus* reported here were 3.6- and 3.8-fold higher, respectively, compared to that reported in a previous clinical trial^6^. This observed discrepancy in ivermectin-associated mosquito mortality can be explained by the cumulative mosquito-lethal effects of ivermectin and metabolites (*i.e.,* M1, M3, and M6) present in human blood post treatment whereas ivermectin-spiked blood only contains the parent compound. It should be noted that the previous clinical trial administered ivermectin at the recommended single dose of 400 µg/kg^6^, which is safe and well-tolerated in MDAs and with repeated dosing^5^, and future pharmacokinetic-pharmacodynamic analyses will assess concentrations of metabolites produced at this dose.

This conclusion differs from a previous study in Kenya which investigated the mosquito-lethal effect of ivermectin co-administered with dihydroartemisinin-piperaquine on *An. gambiae.* The trial concluded that the mosquito killing effect of ivermectin could be described without taking any unknown active metabolites into consideration^8^. While there is a clear concentration–response relationship for ivermectin and mosquito killing, if the active metabolites are not taken into consideration, the overall mosquito-lethal effects are underestimated by a factor of threefold to fourfold. The present study in Thailand clearly demonstrates that the primary human ivermectin metabolites have mosquito-lethal properties that confer clinically relevant effects. It seems very likely that these results are generalizable but further studies with other mosquitoes and disease vectors should be conducted to characterize metabolite activities.

The primary metabolites found in livestock (*e.g.*, cattle, pigs, sheep) tissues are hydroxylation at C-24 (24-hydroxymethyl ivermectin) and 3-O”-demethylation (*i.e.*, M1)^11–13^. It is possible that there are different ivermectin metabolites circulating in livestock blood post-treatment and this should be evaluated further. The subcutaneous route for veterinary ivermectin leads to slower elimination compared to oral administration to humans, which could also alter the exposures of these metabolites in blood over time. There are important pharmacokinetic differences between animals, for example the elimination of ivermectin from pigs is much more rapid compared to cattle, leading to much lower total bioavailability even with the higher standard 300 µg/kg dose in pigs compared to 200 µg/kg dose in cattle^14^. A full investigation into the mosquito-lethal pharmacokinetic-pharmacodynamic effect of ivermectin-treated livestock and the potential contribution of metabolites is warranted.

The main limitation of this study is that non-specific binding of ivermectin and its metabolites to plastic was a confounder. Non-specific binding of ivermectin to plastics has been shown to occur previously with reported recoveries lower than 30%^15^, which is similar to our drug dilution evaluations. This suggests that the 20- and 35-fold differences in mosquito-lethal effect from ivermectin-spiked blood compared to blood from treated subjects from our previous publication^6^ are overestimated because of non-specific plastic binding. The relevance of these findings to those from other laboratories depends on the various plastics and reagents used for making dilutions. Ivermectin binds avidly to plasma with 93% affinity^16^, so our current recommendation is to make ivermectin dilutions in plasma (or media containing plasma) and glass vials. For these reasons we used the measured concentrations and not the added concentrations in these concentration-effect assessments.

Mathematical modelling is increasingly influential in planning malaria control and elimination policies. Utility depends on both the structure and generalizability of the models and the accuracy of the input parameters. Exercises to predict the impact of ivermectin on malaria need to accommodate the prolonged mosquito-lethal activity resulting from its metabolites.

These in vitro results demonstrate that the three primary ivermectin metabolites found in humans (M1, M3, and M6) have a mosquito-lethal effect in *An. dirus* and *An. minimus,* two primary malaria vectors in the GMS. The ivermectin 10-day-LC_50_ values associated with ivermectin-spiked blood compared to blood collected from treated volunteers were 3.6-fold and 3.8-fold higher (*i.e.*, mosquito killing was lower) for *An. dirus* and *An. minimus*, respectively. This large difference in mosquito-lethal effect is fully explained by the contribution of ivermectin metabolites. Population pharmacokinetic-pharmacodynamic modelling is needed to understand the concentration–time profiles of ivermectin and its metabolites, and their relationship to mosquito mortality, to fully predict the duration and magnitude of ivermectin clinical mosquito lethal effects.

## Methods

### Metabolite production

Powdered ivermectin parent compound was obtained from Sigma-Aldrich (St. Louis, Missouri, USA). The M3 (4-hydroxymethyl ivermectin) metabolite was synthesized by synthetically modifying the ivermectin parent compound (WuXi AppTec (Tianjin) Co., Ltd, Tianjin, China). The M1 (3″-*O*-demethyl ivermectin) metabolite was generated by exposure of ivermectin parent compound to biotransformation by a proprietary bacteria strain (Hypha ID: Sp159) at Hypha Discovery. First, a screening process was performed wherein ivermectin parent compound was subjected to whole-cell biotransformations using different bacterial species and strains known to produce oxidized metabolites of exogenous compounds. The bacterial culture extracts were shipped to MORU for evaluation by LC–MS/MS to identify which strain produced only the target M1 structure. The LC–MS/MS system used was an ultra-high performance liquid chromatography (Agilent 1260 Quaternary pump, Agilent 1260 High Performance autosampler, and Agilent 1290 Thermostatted Column Compartment SL, Agilent Technologies) coupled to a quadrupole time-of-flight mass spectrometer (Q-TOF–MS) (TripleTOF 5600+, Sciex) with an electrospray ionization using a DuoSpray ion source^9^. Once the ideal bacterial strain for production of M1 was identified, then bacterial biotransformation was scaled up in one 2L batch exposed to 200 mg of ivermectin (*i.e.*, 100 μg/mL) which, after extraction and purification, produced a final quantity of 41.8 mg of the M1 metabolite. No bacterial strains were identified which produced M6 (3″-*O*-demethyl, 4-hydroxymethyl ivermectin) in sufficient, tractable quantities directly from ivermectin. Therefore, an additional lot of M3 was synthesized and 200 mg was biotransformed using 2 × 1L batches of the same bacterial strain used to produce M1. This resulted in 6.0 mg of the extracted and purified M6 metabolite. Identities of the M1, M3 and M6 metabolite products were confirmed by near magnetic resonance (NMR) spectroscopy. Further information on these processes and structural elucidation aspects are provided in the Supplementary Materials.

### Ethics approval and consent to participate

The study protocol was approved by the ethics committees of the Faculty of Tropical Medicine, Mahidol University (MUTM-2018-069-01), the Oxford University Tropical Research Ethics Committee (OXTREC 33-18), and the Walter Reed Army Institute of Research (WRAIR#2609). This study is registered on ClinicalTrials.gov as NCT03690453. All experiments were performed in accordance with the relevant guidelines and regulations of the above listed institutions. Each blood donor volunteer was provided with an explanation of the study and signed a written informed consent before study entry.

### Mosquito blood meal preparation

Whole blood was collected from healthy volunteers on the day of each mosquito membrane feed. Blood was drawn into sodium heparin tubes. Compounds were dissolved in dimethylsulfoxide (DMSO) to concentrations of 2 mg/mL and 12 μL aliquots were frozen at − 20 °C. Compounds were thawed and serial dilutions were made in phosphate buffered saline (PBS) with 10 μL added to 990 μL of blood to reach final concentration desired for mosquito membrane feeding assays. Control blood meals consisted of previously frozen DMSO diluted in PBS to match the highest ratio of DMSO and PBS fed to mosquitoes in the compound-containing blood meals. A 50 µL aliquot of each mosquito blood meal was frozen at − 80 °C for LC–MS/MS analysis.

### Mosquitoes

All mosquitoes were reared at the Armed Forces Research Institute of Medical Sciences Department of Entomology in Bangkok, Thailand. *Anopheles dirus* s.s. and *An. minimus* s.s. were produced as described previously^17^. Adult mosquitoes used for experiments were provided 10%sucrose solution ad libitum. Mosquitoes were reared at 25 ± 2 °C and 80 ± 10% relative humidity, and a 12 h light:12 h dark photoperiod. Mosquitoes were between 5 and 7 days post emergence at time of blood feeding, and were sugar-starved with access to water from 12 to 18 h before their blood meal.

### Mosquito membrane feeding and mortality assays

At each mosquito membrane feed 1 mL of whole blood mixed with the different compounds over a range of concentrations were offered to groups of 40 *An. dirus* and 40 *An. minimus* mosquitoes via membrane feeders warmed to 37 °C. After feeding, up to 30 blood-fed mosquitoes of each species were gently transferred via aspiration to clean cardboard containers (0.5 L). After the blood meal, mosquitoes were maintained in an incubator at 25 ± 1 °C and 80 ± 10% humidity, and offered 10% sucrose ad libitum. Mosquito survival was monitored daily for fourteen days and any dead mosquitoes were removed by aspiration and recorded. Fourteen days after the blood meal any remaining mosquitoes were recorded as alive and then frozen. The LC_50_ and LC_90_ of mosquitoes were estimated using a normalized concentration–response analysis (IC_50_ and Hill), assuming a maximum of 100% mosquito mortality and an estimated baseline mosquito mortality (*i.e.*, mosquito mortality at zero drug concentration). The median time to death were calculated for mosquitoes subjected to 1 ± 0.5 ng/mL and 5 ± 0.5 ng/mL of ivermectin, using both clinical data and in vitro data. The median time to death for each unique ivermectin concentration was also calculated and data evaluated with a normalized concentration–response analysis (TC_50_ and Hill), assuming a maximum time to mosquito mortality of 10 days and an estimated baseline rate of mosquito mortality (*i.e.*, rate of mosquito mortality at zero drug concentration). A similar analysis was conducted when comparing median time to death for ivermectin and its metabolites, assuming a maximum time to mosquito mortality of 14 days and a fixed hill-slope of an average value of -2.0 to facilitate model fitting. All mosquito survival analyses were performed with GraphPad Prism v.9.0 (GraphPad Software Inc, San Diego, CA, USA).

### Ivermectin and metabolite quantification by LC–MS/MS

Samples (100 µL) was aliquoted into a 1-mL 96-well extraction plate followed by the addition of cold extraction solution (450 µL of 90% acetonitrile in water containing 15 ng/mL internal standard ivermectin-d2) into each well using a multi-dispenser pipette. The mixture was mixed on a MixMate set to 1000 rpm for 10 min. The suspension was then centrifuged at 2100×*g* for 10 min at 4 °C to separate the precipitates. The clear supernatant (380 µL) was transferred onto a HybridSPE® Plus 96-well plate (P/N 575659-U, Supelco, Darmstadt, Germany) to remove both precipitated proteins and phospholipids. 10 mM ammonium formate in water containing 0.1% formic acid (100 µL) was used to extract the sample compounds into the receiving plate. The final extract was mixed at 1000 rpm for 5 min and centrifuged at 1100×*g* for 5 min at 4 °C before the LC–MS/MS analysis.

LC–MS/MS analysis was carried out using a Thermo Scientific (Germering, Germany) Dionex Ultimate 3000 UHPLC system, coupled with Sciex (Woodlands, Singapore) triple quadrupole 6500 + mass spectrometer. The Ultimate 3000 UHPLC was equipped with an Acquity HSS T3 (2.1 × 100 mm, 1.8 µm, Waters (Milford, MA)) with a 5 mm Vanguard pre-column (Waters) of the same internal diameter, particle size, and type. Flow rate was maintained at 0.5 mL/min. The mobile phase contained (A) 10 mM ammonium formate in ultrapure water and (B) 5:95 (v/v) 10 mM ammonium formate in water/acetonitrile, both A and B with 0.1% formic acid. Gradient elution started at 75% B, ramped to 90% B in 3 min, and held constant for 6 min. The gradient returned to 75% B in 0.1 min with 2 min re-equilibration until the next injection. A divert valve switched flow from waste to the MS/MS after 2.4 min until 7 min. The total run time from injection to injection was approximately 11 min. The autosampler tray was set at 4 °C, column temperature was 40 °C, and injection volume was 5 µL.

Electrospray ionization (ESI) operated in positive mode and multiple reaction monitoring (MRM) was used in MS/MS. Three transitions were monitored for each targeted analyte^9^. Dwell time was set at 0.15 s to yield greater than six points for each chromatographic ion peak. Table S3 lists the MS/MS conditions for each analyte in order of retention times (*t*_R_). Curtain gas setting was 30 psi, source gases 1 and 2 settings were 40 psi and 55 psi, respectively, medium collision gas setting with ion spray voltage at + 5000 V, and the source temperature gas setting was 225 °C. Sciex Analyst 1.7.2 software (Woodlands, Singapore) was used for both instrument control data processing.

The LLOQ was determined for each analyte combination based on accuracy and precision data after samples were taken through the entire workflow including sample extraction and LC–MS/MS analysis. LLOQs were typically 0.25 ng/mL for all targeted analytes.

All determinations for individual ivermectin, M1 and M3 metabolites were calibrated using multipoint, calibration standards in whole blood, which bracketed the quality control (QC) samples. QC samples of ivermectin, M1 and M3 at four concentration levels were processed (triplicates at each level) simultaneously with the mosquito-blood meal samples and measured in a single analytical series, in order to ensure the overall method performance and the quality of reported drug concentrations. Due to the limitation of M6 reference standard material amounts available at the time of analysis, the calibration curve and other internal quality control parameters of the ivermectin parent drug were used to assess concentration measurements of M6.

Analyte identification in sample extracts was accomplished via *t*_R_ and the presence of monitoring trace MRMs.

## Supplementary Information

Supplementary Information.

## Data Availability

The datasets generated during the current study are available from the corresponding author on reasonable request.
