# Author Correction: Ivermectin metabolites reduce *Anopheles* survival

**DOI:** 10.1038/s41598-024-76902-z

**Published:** 2024-10-24

**Authors:** Kevin C. Kobylinski, Phornpimon Tipthara, Narenrit Wamaket, Sittinont Chainarin, Rattawan Kullasakboonsri, Patchara Sriwichai, Siriporn Phasomkusolsil, Borimas Hanboonkunupakarn, Podjanee Jittamala, Renia Gemmell, John Boyle, Stephen Wrigley, Jonathan Steele, Nicholas J. White, Joel Tarning

**Affiliations:** 1 Department of Entomology, Armed Forces Research Institute of Medical Sciences, 315/6 Rajvithi Road, Bangkok, 10400 Thailand; 2 Mahidol Oxford Tropical Medicine Research Unit, Faculty of Tropical Medicine, Mahidol University, 420/6 Rajvithi Road, Ratchathewi, Bangkok, 10400 Thailand; 3 Mahidol Vivax Research Unit, Faculty of Tropical Medicine, Mahidol University, 420/6 Rajvithi Road, Ratchathewi, Bangkok, 10400 Thailand; 4 Department of Medical Entomology, Faculty of Tropical Medicine, Mahidol University, 420/6 Rajvithi Road, Ratchathewi, Bangkok, 10400 Thailand; 5 Department of Clinical Tropical Medicine, Faculty of Tropical Medicine, Mahidol University, 420/6 Rajvithi Road, Ratchathewi, Bangkok, 10400 Thailand; 6 Department of Tropical Hygiene, Faculty of Tropical Medicine, Mahidol University, 420/6 Rajvithi Road, Ratchathewi, Bangkok, 10400 Thailand; 7 Hypha Discovery Limited, 154B Brook Drive, Abingdon, Oxfordshire OX14 4SD UK; 8 Centre for Tropical Medicine and Global Health, Nuffield Department of Clinical Medicine, University of Oxford, Old Road Campus, Oxford, OX3 7BN UK

Correction to: *Scientific Reports*10.1038/s41598-023-34719-2, published online 19 May 2023

The original version of this Article contained errors in the Methods section, under the subheading ‘Ethics approval and consent to participate’.

 “The study protocol was approved by the ethics committees of the Faculty of Tropical Medicine, Mahidol University (MAL18006), the Oxford University Tropical Research Ethics Committee (OXTREC 33-18), and the Walter Reed Army Institute of Research (WRAIR#2609). All experiments were performed in accordance with the relevant guidelines and regulations of the above listed institutions. Each blood donor volunteer was provided with an explanation of the study and signed a written informed consent before study entry.”

now reads:

“The study protocol was approved by the ethics committees of the Faculty of Tropical Medicine, Mahidol University (MUTM-2018-069-01), the Oxford University Tropical Research Ethics Committee (OXTREC 33-18), and the Walter Reed Army Institute of Research (WRAIR#2609). This study is registered on ClinicalTrials.gov as NCT03690453. All experiments were performed in accordance with the relevant guidelines and regulations of the above listed institutions. Each blood donor volunteer was provided with an explanation of the study and signed a written informed consent before study entry.”

The original Article has been corrected.

