# Supplementary material for: Ivermectin metabolites reduce Anopheles survival
Source: Sci Rep. 2023 May 19;13:8131. doi: 10.1038/s41598-023-34719-2 (PMC10199058; doi:10.1038/s41598-023-34719-2)
Supplement: Supplementary file 1 — Supplementary Information. [file 41598_2023_34719_MOESM1_ESM.docx]

# Supplemental Material

# Production of M1 (3”-*O*-demethyl ivermectin)

## Scaled-up fermentation

Scalable production of M1 was confirmed via proprietary microbial strain (Hypha ID: Sp159), with optimal production observed when dosed with the ivermectin substrate 48 hours after inoculation into production media and anticipated optimal harvest after a further 120 to 144 hours. The total fermentation volume of 2L was completed as a single fermentation produced as follows: sufficient Sp159 starter cultures were prepared by inoculating Erlenmeyer flasks (250 ml flasks containing 50 mL of proprietary M3G medium) with 100 µL of Sp159 (from cryo-preserved glycerol stocks stored in liquid nitrogen) then incubating for 3 days at 27°C and 200 rpm. After the specified growth period, 40 x 250 ml Erlenmeyer flasks, each of which contained 50 mL of M3G medium, were each inoculated with 1 mL seed culture and incubated at 27°C and 200 rpm. After 48 hours, all cultures were dosed with 1mg/ml ivermectin formulated in 60% w/v HP-*β*-CD to provide a final concentration of 100 mg/L and 6% w/v HP-*β*-CD. Five flasks were selected at random and sampled daily with the reaction harvested based upon the total peak area detected for M1 at UV_254nm_ 121.5 hours after dosing.

## Purification

The fermentation was centrifuged and the supernatant extracted twice with an equivalent volume of EtOAc. The biomass was also extracted with 1 L EtOAc. The organic layers were combined and dried under vacuum to afford an orange oil. This oil was dissolved in DMSO (15 mL) and fractionated over a Waters XBridge BEH C18 column (30 x 100 mm + 30 x 10 mm guard column) eluting with a flow rate of 40 mL/min as follows; the gradient started at 35/60/5% (H_2_O/MeCN/2% v/v formic acid_(aq)_) and increased to 15/80/5% over 22 minutes, followed by wash and re-equilibration steps. M1 eluted between 14.8 and 16.0 minutes with subsequent pooling, drying under vacuum and lyophilisation yielding 41.8 mg of a white powder at >95% purity by LC-UV-ELSD.

## Structure Elucidation by NMR Spectroscopy

Near magnetic resonance (NMR) spectra were acquired on a 700 MHz instrument. Standard methods were used to acquire ^1^H, COrrelated SpectroscopY (COSY), Heteronuclear Single Quantum Coherence (HSQC) and Heteronuclear Multiple Bond Correlation (HMBC) NMR spectra. The ^1^H, COSY, HSQC, and HMBC spectra of the parent compound, ivermectin B_1a_ in chloroform (CDCl_3_) are shown in Figures S1-4, respectively. The assignment of the ^1^H and ^13^C NMR signals to the structure was straightforward and these signal assignments are summarised in Table S1.

The ^1^H, COSY, HSQC, and HMBC spectra of M1 are shown in Figures S5-8, respectively. As expected, comparison of its ^1^H and HSQC spectra with those of ivermectin B1a indicated that only one methoxyl group was present with signals at δ_H_/δ_C_ 3.42/56.5 ppm compared to two such groups for ivermectin B_1a_ with signals at 3.43/56.4 and 3.42/56.4 ppm. The M1 methoxyl proton signal shared an HMBC correlation with a carbon signal at 79.4 ppm, suggesting that it was attached at the 3’-position, and the other proton and carbon signals around this ring were very similar to those of ivermectin B_1a_. A new methine signal at δ_H_/δ_C_ 3.90/69.2 ppm was apparent in the M1 spectra and this showed HMBC correlations with carbon signals at 78.2 and 38.2 ppm, corresponding to a methine group with a proton signal at 3.12 ppm and a methylene group with proton signals at 2.12 and 1.70 ppm. COSY correlations confirmed that the proton signals at 2.12/1.70, 3.90 and 3.12 ppm formed a contiguous system consistent with positions 2’’, 3’’ and 4’’, respectively. The corresponding carbon signals for these positions were all shifted compared to those for ivermectin B_1a_, confirming that M1 was formed by *O*-demethylation at the 3’’-position. The ^1^H and ^13^C NMR signal assignments for ivermectin B_1a_ M1 metabolite sample are summarised in Table S1.

**Table S1. ^1^H and ^13^C NMR signal assignments for ivermectin B_1a_ and M1 metabolite.**

| **Position** | **Ivermectin B_1a_** | | **M1 metabolite** | |
| --- | --- | --- | --- | --- |
|  | **δ_C_/ppm** | **δ_H_^†^/ppm, m (J/Hz)** | **δ_C_/ppm** | **δ_H_^†^/ppm, m (J/Hz)** |
| 1  2  3  4  4a  5  6  7  8  8a  9  10  11  12  12a  13  14  14a  15  16  17  18  19  20  21  22  23  24  24a  25  26  26a  27  28  1’  2’  3’  3’-OCH3  4’  5’  6’  1’’  2’’  3’’  3’’-OCH3  4’’  5’’  6’’ | 173.9  45.8  118.0  138.0  20.0  67.7  79.1  80.4  139.7  68.5  120.5  124.7  138.1  39.8  18.4  81.8  135.0  15.2  118.4  34.2  67.2  37.0  68.6  41.2  97.5  35.8  28.1  31.2  17.5  76.8  35.6  12.1  27.3  12.4  94.8  34.5  79.4  56.4  80.5  67.2  18.4  98.5  34.1  74.2  56.4  76.2  68.1  17.7 | -  3.28, q (2.2)  5.42, bs  -  1.87, s  4.29, t (6.8)  3.96, d (6.3)  -  -  4.69, dd (14.3, 2.4); 4.66, dd (14.2, 2.1)  5.86, dt (10.3, 2.1)  5.71, dd (15.1, 10.4)  5.75, dd (15.1, 9.1)  2.51, m  1.16, d (6.9)  3.94, bs  -  1.49, s  4.98, d (11.2)  2.27, m; 1.52, m  3.67, m  1.76, m; 0.82, m  5.35, tt (11.2, 4.8)  1.97, dd (12.3, 3.5); 1.35, t (11.8)  -  1.66, m; 1.48, m  1.52, m  1.52, m  0.78, d (5.8)  3.21, dd (9.3, 1.2)  1.56, m  0.85, d (6.7)  1.46, m; 1.42, m  0.93, t (7.4)  4.77, d (3.5)  2.22, dd (12.7, 4.8); 1.56, m  3.62, ddd (11.4, 8.7, 4.7)  3.43, s  3.24, t (9.1)  3.82, dq (9.1, 6.3)  1.25, d (6.6)  5.39, d (3.7)  2.33, dd (12.3, 4.3); 1.53, m  3.48, ddd (11.5, 9.0, 4.8)  3.42, s  3.16, t (9.1)  3.76, dq (9.2, 6.2)  1.27, d (6.3) | 173.8  45.8  118.1  137.9  20.0  67.8  79.1  80.4  139.6  68.5  120.5  124.7  138.1  39.8  20.2  81.4  135.0  15.1  118.3  34.4  67.2  37.0  68.7  41.3  97.5  35.8  28.2  31.3  17.5  76.7  35.5  12.2  27.2  12.5  94.7  34.6  79.4  56.5  80.7  67.2  18.5  98.4  38.2  69.2  -  78.2  68.2  17.6 | -  3.28, m  5.42, bs  -  1.87, s  4.29, d (5.9)  3.97, d (6.1)  -  -  4.69, dd (14.4, 1.5); 4.66, dd (14.5, 1.5)  5.86, d (10.2)  5.71, dd (14.9, 10.3)  5.74, dd (14.9, 9.4)  2.51, m  1.14, d (6.9)  3.93, bs  -  1.49, s  4.98, d (10.9)  2.22, m; 1.55, m  3.66, m  1.76, dd (12.0, 2.8); 0.82, m  5.34, tt (10.8, 4.8)  1.98, dd (12.1, 4.7); 1.35, t (11.9)  -  1.65, m; 1.48, m  1.52, m  1.52, m  0.78, d (5.7)  3.21, d (8.9)  1.55, m  0.85, d (6.8)  1.46, m; 1.41, m  0.93, t (7.4)  4.77, d (3.4)  2.22, m; 1.56, m  3.61, m  3.42, s  3.23, t (8.8)  3.82, dq (9.6, 6.3)  1.25, d (6.4)  5.35, d (3.0)  2.21, m; 1.70, td (12.5, 3.8)  3.90, m  -  3.12, t (9.0)  3.74, dq (9.6, 6.3)  1.27, d (6.3) |

^†^Referenced to chloroform at 7.260 ppm; bs = broad singlet; s = singlet; d = doublet; t = triplet; q = quartet; m = multiplet.

**Figure S1. 700 MHz ^1^H NMR spectrum of ivermectin in CDCl_3_.**

**Figure S2. COSY NMR spectrum of ivermectin in CDCl_3_.**

**Figure S3. HSQC NMR spectrum of ivermectin in CDCl_3_.**

**Figure S4. HMBC NMR spectrum of ivermectin in CDCl_3_.**

**Figure S5. 700 MHz ^1^H NMR spectrum of M1 metabolite sample in CDCl_3_.**

**Figure S6. COSY NMR spectrum of M1 metabolite sample in CDCl_3_.**

**Figure S7. HSQC NMR spectrum of M1 metabolite sample in CDCl_3_.**

**Figure S8. HMBC NMR spectrum of M1 metabolite sample in CDCl_3_.**

**Figure S9. Spectra of M1 metabolite using shallow gradient analysis.**

From top to bottom, the spectra are positive ion ESI-MS and negative ion ESI-MS.

Major positive ion MS peaks; 861.5m/z [M+H]^+^, 878.5m/z [M+NH_4_]^+^, 883.4m/z [M+Na]^+^, 843.4m/z [M+H-H_2_O]^+^, 825.4m/z [M+H-2xH_2_O]^+^, 811.4m/z [M+H-2xH_2_O-CH_2_]^+^, 793.4m/z [M+H-3xH_2_O-CH_2_]^+^, 731.4m/z [M+H-(C_6_H_11_O_3_)]^+^, 713.4m/z [M+H-(C_6_H_11_O_3_)-H_2_O]^+^, 695.4m/z [M+H-(C_6_H_11_O_3_)-2xH_2_O]^+^, 681.4m/z [M+H-(C_6_H_11_O_3_)-2xH_2_O-CH_2_]^+^, 663.3m/z [M+H-(C_6_H_11_O_3_)-3xH_2_O-CH_2_]^+^, 645.3m/z [M+H-(C_6_H_11_O_3_)-4xH_2_O-CH_2_]^+^, 587.3m/z [M+H-(C_13_H_23_O_6_)]^+^, 569.3m/z [M+H-(C_13_H_23_O_6_)-H_2_O]^+^ and 551.3m/z [M+H-(C_13_H_23_O_6_)-2xH_2_O]^+^.

Major negative ion MS peaks; 859.6m/z [M-H]^-^, 905.6m/z [M+formic acid-H]^-^ and 973.6m/z [M+TFA-H]^-^.

# Production of M6 (3”-*O*-demethyl, 4-hydroxymethyl ivermectin)


## Scaled-up fermentation

Scalable production of M6 was confirmed via proprietary microbial strain (Hypha ID: Sp159). The total biotransformation volume of 2L was completed via two identical 1 L fermentations produced as follows on subsequent weeks: sufficient Sp159 starter cultures were prepared by inoculating Erlenmeyer flasks (250 mL containing 50 mL of proprietary M3G medium) with 100 µL of Sp159 (from cryo-preserved glycerol stocks stored in liquid nitrogen) then incubating for 3 days at 27°C and 200 rpm. After the specified growth period, 20 x 250 mL Erlenmeyer flasks, each of which contained 50 mL of M3G medium, were each inoculated with 1 mL seed culture and incubated at 27°C and 200 rpm. After 48 hours, all cultures were dosed with 1 mg/mL M3 (derived from chemical synthesis) pre-formulated in 60% w/v HP-*β*-CD to provide a final concentration of 100 mg/L and 6% w/v HP-*β*-CD. Five flasks were selected at random and sampled daily with the reactions harvested based upon the total peak area detected for ivermectin-M6 at UV_254nm_. Batches 1 and 2 were harvested 54 and 122 hours after dosing, respectively, based on time-course sample analysis observations.

## Purification

The M6-containing biotransformation fermentation broth was centrifuged and the supernatant extracted twice with an equivalent volume of ethyl acetate. The biomass was also extracted with EtOAc (500 mL), centrifuged, and the pellet re-extracted with 1:8 Me_2_CO/EtOAc before further centrifugation. The organic layers were dried over anhydrous magnesium sulfate, filtered and the solvent removed *in vacuo* to afford an orange residue. The residue was dissolved in DMSO (5 mL) and fractionated over a Waters XBridge Prep C18 5 µm OBD column (30 × 100 mm + 30 × 10 mm guard column) eluting with a flow rate of 40 mL/min as follows; the gradient started at 60/35/5% (H_2_O/MeCN/2% v/v formic acid_(aq)_) was held for 1 minute, then increased to 40/55/5% over 22 minutes (t = 23 minutes) followed by wash and re-equilibration steps. M6 eluted between 16 and 17 minutes with subsequent pooling, drying *in vacuo* to afford a white powder. The product contained a significant unknown, unrelated impurity. Therefore the powder was dissolved in a minimal volume of DMSO and fractionated on the same column as above with a gradient held at 90/5/5% (H_2_O/MeCN/2% v/v formic acid_(aq)_) for 4 minutes, then increased to 60/35/5% over 1 minute (t = 5 minutes) and increased again to 40/55/5% over 22 minutes (t = 27 minutes) followed by wash and re-equilibration steps. M6 eluted between 14.2 and 15.2 minutes with subsequent pooling, drying *in vacuo* and lyophilisation yielding 6.00 mg of M6 metabolite as a white powder at >90% purity by LC-UV-ELSD.

## Structure Elucidation by NMR Spectroscopy

NMR spectra were acquired at the University of Bristol on a Bruker AVANCE III HD 700 NMR spectrometer equipped with a 1.7 mm microcryoprobe. Standard methods were used to acquire ^1^H, COSY, HSQC and HMBC NMR spectra. Carbon shifts were detected indirectly *via* heteronuclear shift correlation spectroscopy (HSQC and HMBC spectra). The ^1^H, COSY, HSQC, and HMBC spectra of the M6 metabolite sample in CDCl_3_ are shown in Figures S10-13, respectively. As for the previous M1 metabolite, inspection of its ^1^H and HSQC spectra indicated that only one methoxyl group was present with signals at δ_H_/δ_C_ 3.42/56.6 ppm. This methoxyl proton signal shared an HMBC correlation with a carbon signal at 79.4 ppm, suggesting that it was attached at the 3’-position, and the other proton and carbon signals around this ring were almost identical to those of ivermectin B_1a_ and the M1 metabolite. As in the spectra for M1 compared to those for ivermectin B_1a_, the spectra of M6 showed the presence of a new methine signal at δ_H_/δ_C_ 3.90/69.2 ppm, and this showed HMBC correlations with carbon signals at 78.1 and 38.1 ppm. The other signals associated with this ring system for JB89/100/4 were very similar to those observed for M1, supporting *O*-demethylation at the 3’’-position. Further comparison of the spectra of M6 with those for ivermectin B_1a_ itself indicated that the signals for the methyl group at position 4a were absent. In their place were two methylene doublet proton signals at 4.31 and 4.25 ppm, sharing an associated carbon shift of 64.9 ppm. These proton signals showed HMBC correlations with carbon signals at 120.2, 140.1 and 65.8 ppm, corresponding to positions 3, 4 and 5, respectively. This is consistent with hydroxylation at position 4a. M6 metabolite was therefore confirmed to be the expected metabolite: 3”-*O*-demethyl-4-hydroxymethylivermectin. Its ^1^H and ^13^C NMR signal assignments are detailed in Table S2.

**Table S2. ^1^H and ^13^C NMR signal assignments for M6 metabolite.**

| **Position** | **M6** | | **Position** | **M6** | |
| --- | --- | --- | --- | --- | --- |
|  | **δ_C_/ppm** | **δ_H_^†^/ppm, m (J/Hz)** |  | **δ_C_/ppm** | **δ_H_^†^/ppm, m (J/Hz)** |
| 1  2  3  4  4a  5  6  7  8  8a  9  10  11  12  12a  13  14  14a  15  16  17  18  19  20 | 173.4  45.6  120.2  140.1  64.9  65.8  78.9  80.6  138.9  68.4  120.8  124.7  138.4  39.9  20.3  81.7  135.0  15.1  118.2  34.0  67.2  37.1  68.8  49.2 | -  3.34, bs  5.74, bs  -  4.31, d (13.0); 4.25, d (13.0)  4.59, d (4.9)  3.99, d (6.1)  -  -  4.71, d (14.4); 4.68, d (14.5)  5.88, d (10.4)  5.72, dd (15.1, 10.7)  5.76, dd (15.1, 9.6)  2.51, m  1.15, d (6.7)  3.94, bs  -  1.50, s  4.99, d (10.7)  2.28, t (12.4); 1.56, m  3.67, td (10.9, 2.7)  1.77, dd (11.3, 2.4); 0.82, m  5.35, tt (11.0, 5.0)  1.98, dd (12.3, 4.3); 1.35, t (11.8) | 21  22  23  24  24a  25  26  26a  27  28  1’  2’  3’  3’-OCH3  4’  5’  6’  1’’  2’’  3’’  3’’-OCH3  4’’  5’’  6’’ | 97.5  35.8  28.1  31.3  17.5  76.7  35.8  12.2  27.3  12.5  94.7  34.4  79.3  56.6  80.7  67.2  18.5  98.4  38.1  69.2  -  78.1  68.1  17.5 | -  1.65, d (12.5); 1.49, m  1.52, m  1.52, m  0.79, d (5.6)  3.22, m  1.55, m  0.86,d (6.7)  1.46, m; 1.42, m  0.93, t (7.4)  4.78, d (3.0)  2.21, m; 1.56, m  3.61, m  3.42, s  3.23, m  3.82, dq (8.9, 5.9)  1.25, d (6.1)  5.35, d (3.4)  2.21, m; 1.71, td (12.3, 3.8)  3.90, m  -  3.13, t (9.1)  3.74, dq (9.2, 6.2)  1.27, d (6.1) |

^†^Referenced to chloroform at 7.260 ppm; bs = broad singlet.

**Figure S10. 700 MHz ^1^H NMR spectrum of M6 metabolite in CDCl_3_.**

**Figure S11. COSY NMR spectrum of M6 metabolite in CDCl_3_.**

**Figure S12. HSQC NMR spectrum of M6 metabolite in CDCl_3_.**

**Figure S13. HMBC NMR spectrum of M6 metabolite in CDCl_3_.**

**Figure S14. Spectra of M6 metabolite using shallow gradient analysis.**

From top to bottom the spectra are positive ion ESI-MS and negative ion ESI-MS.

Major positive ion MS peaks; 877.8m/z [M+H]^+^, 894.8m/z [M+NH_4_]^+^, 899.8m/z [M+Na]^+^, 859.8m/z [M+H-H_2_O]^+^, 841.8m/z [M+H-2xH_2_O]^+^, 729.7m/z [M+H-(C_6_H_11_O_4_)-H_2_O]^+^, 711.7m/z [M+H-(C_6_H_11_O_4_)-2xH_2_O]^+^, 697.6m/z [M+H-(C_6_H_11_O_4_)-2xH_2_O]^+^, 679.6m/z [M+H-(C_6_H_11_O_4_)-3xH_2_O]^+^, 585.6m/z [M+H-(C_13_H_23_O_7_)]^+^, 567.5m/z [M+H-(C_13_H_23_O_7_)-H_2_O]^+^ and 549.5m/z [M+H-(C_13_H_23_O_7_)-2xH_2_O]^+^.

Major negative ion MS peaks; 875.9m/z [M-H]^-^, 921.9m/z [M+formic acid-H]^-^, 990.0m/z [M+TFA-H]^-^, 857.9m/z [M-H_2_O-H]^-^ and 839.9m/z [M-2xH_2_O-H]^-^**.**

**Figure S15: Spectra of M3 metabolite using shallow gradient analysis.**

From top to bottom the spectra are positive ion ESI-MS and negative ion ESI-MS.

Major positive ion MS peaks; 891.7m/z [M+H]^+^, 908.8m/z [M+NH_4_]^+^, 873.7m/z [M+H-H_2_O]^+^, 855.7m/z [M+H-2xH_2_O]^+^, 823.6m/z [M+H-3xH_2_O-CH_2_]^+^, 729.6m/z [M+H-(C_7_H_13_O_4_)]^+^, 711.6m/z [M+H-(C_7_H_13_O_4_)-H_2_O]^+^, 693.6m/z [M+H-(C_7_H_13_O_4_)-2xH_2_O]^+^, 585.5m/z [M+H-(C_14_H_25_O_7_)]^+^, 567.5m/z [M+H-(C_14_H_25_O_7_)-H_2_O]^+^ and 549.5m/z [M+H-(C_14_H_25_O_7_)-2xH_2_O]^+^.

Major negative ion MS peaks; 889.9m/z [M-H]^-^, 935.9m/z [M+formic acid-H]^-^ and 1003.9m/z [M+TFA-H]^-^.

**Table S3. MS/MS conditions in the analysis of ivermectin and metabolites.**

| **Analyte** | ***t*_R_**  **(min)** | **Precursor ion (m/z)** | **Product ion (m/z)** | **DP (V)** | **EP (V)** | **CE (V)** | **CXP (V)** |
| --- | --- | --- | --- | --- | --- | --- | --- |
| M6 (3”-*O*-demethyl, 4-hydroxymethyl ivermectin) | 2.70 | 894.4 | 307.2  567.3  585.3 | 50 | 10 | 40  30  25 | 35  30  35 |
| M3 (4-hydroxymethyl ivermectin) | 3.80 | 908.5 | 307.2  585.6  567.3 | 50 | 10 | 40  25  30 | 35  35  35 |
| M1 (3”-*O*-demethyl ivermectin) | 4.69 | 878.5 | 307.2  569.4  551.6 | 50 | 10 | 35  20  25 | 35  35  35 |
| Ivermectin-d_2_ | 6.04 | 894.5 | 309.1  571.2 | 50 | 10 | 35  21 | 35  35 |
| Ivermectin | 6.08 | 892.5 | 569.2  307.2  551.2 | 50 | 10 | 21  34  30 | 35  35  35 |

*t*_R_ = retention time; DP = declustering potential; EP = entrance potential; CE = collisional energy; CXP = collision cell exit potential

**Figure S16. Time to median mosquito mortality,comparing *in vivo* clinical data (upper panels) and *in vitro* data (lower panels) for *An. dirus* (left panels) and *An. minimus* (right panels).**

Open circles are observed median time to death during 10 days of observation, within each unique ivermectin concentration evaluated (with the assumption that all alive mosquitoes at day 10 were counted as dead to avoid censoring of data). Data were analysed using a concentration-response analysis, with a maximum time to death of 10 days. The model mean is shown as a solid line with its 95% confidence interval as a shaded area. TC_50_ is the estimated ivermectin concentration (95% confidence interval) associated with half of maximum effect $\left( {TC}_{50}=\frac{Maximum time to death + Minimum time to death}{2} \right)$.

**Figure S17. Time to median *An. dirus* mortality, comparing *in vitro* data for ivermectin and its metabolites.**

Open circles are observed median time to death during 14 days of observations, within each unique concentration evaluated (with the assumption that all alive mosquitoes at day 14 were counted as dead to avoid censoring of data). Data were analysed using a concentration-response analysis, with a fixed maximum time to death of 14 days and a fixed hill-slope of -2.0 to facilitate model fitting. The model mean is shown as a blue line with its 95% confidence interval as a shaded area.

TC_50_ is the estimated ivermectin concentration (95% confidence interval) associated with half of maximum effect $\left( {TC}_{50}=\frac{Maximum time to death + Minimum time to death}{2} \right)$.

**Figure S18.** **Time to median *An. minimus* mortality, comparing *in vitro* data for ivermectin and its metabolites.**

Open circles are observed median time to death during 14 days of observations, within each unique concentration evaluated (with the assumption that all alive mosquitoes at day 14 were counted as dead to avoid censoring of data). Data were analysed using a concentration-response analysis, with a fixed maximum time to death of 14 days and a fixed hill-slope of -2.0 to facilitate model fitting. The model mean is shown as a blue line with its 95% confidence interval as a shaded area.

TC_50_ is the estimated ivermectin concentration (95% confidence interval) associated with half of maximum effect $\left( {TC}_{50}=\frac{Maximum time to death + Minimum time to death}{2} \right)$.

**Table S4. *An. dirus* and *An. minimus* TC_50_ values for ivermectin and metabolites calculated based on 14 day observations**

| Species | Compound | TC_50_ [95% CI] (nM) |
| --- | --- | --- |
| *An. dirus* | Ivermectin | 17.0 [12.73 – 25.48] |
|  | M1 | 14.74 [9.55 - 22.00] |
|  | M3 | 13.73 [9.73 - 19.19] |
|  | M6 | 9.63 [5.79 - 15.71] |
| *An. minimus* | Ivermectin | 2.04 [1.47 - 2.77] |
|  | M1 | 2.47 [1.94 - 3.12] |
|  | M3 | 2.48 [1.94 - 3.18] |
|  | M6 | 2.25 [1.67 - 3.00] |
